# Bariatric and Cosmetic Surgery in People with Eating Disorders

**DOI:** 10.3390/nu12092861

**Published:** 2020-09-18

**Authors:** Charmaine D’Souza, Phillipa Hay, Stephen Touyz, Milan K. Piya

**Affiliations:** 1 School of Medicine, Western Sydney University, NSW 2751, Australia; 18715082@student.westernsydney.edu.au (C.D.); m.piya@westernsydney.edu.au (M.K.P.); 2 Camden and Campbelltown Hospitals, South Western Sydney Local Health District, NSW 2560, Australia; 3 School of Psychology and InsideOut Institute, University of Sydney, NSW 2006, Australia; stephen.touyz@sydney.edu.au

**Keywords:** eating disorders, feeding disorders, obesity, bariatric surgery, cosmetic surgery, bulimia nervosa, binge eating disorder

## Abstract

Rates of eating disorders (EDs) are increasing in Australia, as are rates of bariatric and cosmetic surgery including weight-related procedures. It is known that binge eating disorder (BED) is common in bariatric surgery candidates and that people with EDs are likely to undergo weight-related cosmetic procedures, however, most of the literature is based on clinic samples and focuses on young women and BED. Aims of this study were to determine the prevalence of (1) actual or intended bariatric surgery and (2) actual or intended cosmetic surgery including weight-related procedures in people with a current ED and a lifetime history of BED or bulimia nervosa (BN), and the associations with actual or intended bariatric or cosmetic surgery and demographic features. Using a general population survey, 2977 individuals were interviewed regarding sociodemographic status, ED symptoms, mental health-related quality of life (MHRQoL) and actual or intended use of bariatric and cosmetic surgery, prevalence estimates of which were 2.0% and 1.1%, respectively. People who had planned or received either type of surgery were more likely to be (1) women and (2) have a higher BMI, (3) poorer MHRQoL and (4) a current ED, lifetime BN or BED or features of EDs (all *p* < 0.05). Age and household income were not significantly associated with increased use of either type of surgery. Given the potential for an ED to affect outcomes of surgery, screening and treatment for EDs should be considered in such surgical candidates.

## 1. Introduction

Bariatric surgery is an established treatment option for severe obesity and type 2 diabetes mellitus (T2DM), and can result in significant, sustained weight loss and improvement/remission of T2DM [1,2,3,4]. The number of bariatric procedures performed in Australia is increasing with over 20,000 bariatric procedures having been performed in the 2018–2019 financial year [5]. The prevalence of eating disorders (EDs) is also rising and, in Australia, since 1995, ED behaviors have been rising at a higher rate in people with concurrent obesity [6,7]. Anorexia nervosa (AN), bulimia nervosa (BN) and binge-eating disorder (BED) are the main currently recognized EDs [8]. Estimates of their point prevalence in Australia are up to 0.5% for AN broadly defined, 1.2% for BN and 1.5% for BED broadly defined [9]. These figures are similar to international studies [10]. Alongside the rise in EDs are increases in body image concerns in the general population and cosmetic procedures [11,12,13].

Given the increase in EDs and bariatric and other weight-related surgeries, it is important to know if people with EDs are having such surgery and whether they are having this more often than people without an ED. An understanding of the size of the problem may be used to inform bariatric surgery and other clinics. In particular, the presence of BED has been found to be associated with negative outcomes of bariatric surgery. One systematic review found that patients with pre-surgical BED were more likely than not to continue having disordered eating after surgery, including experiencing loss of control while eating, eating while not hungry and feeling “disgusted or distressed” with their eating habits [14]. Persistent BED and ED symptoms post-surgery were also associated with poorer weight outcomes overall, including losing smaller amounts of weight and regaining lost weight [14]. One Brazilian cohort study found that while binge eating, anxiety and depression reduced in the 23 months following bariatric surgery, all three parameters had increased by 60 months following surgery [15]. Studies of night eating syndrome (NES) (an ED with close similarities to BED) reported variable outcomes post-surgery—some where the ED improved and others where it worsened [16].

With regard to the prevalence of EDs in candidates for bariatric surgery, a 2007 systematic review found that rates of BED ranged from 6 to 64%, approximately one to ten times the rate in the general population [14]. Another review found the prevalence of BED in pre-surgical patients was 2% to 53% [17]. Research on the prevalence of other EDs is scarce. To our knowledge, three studies have reported the prevalence of BN in bariatric candidates and found it to range from 2% to 7.6% [18,19,20]. Estimates of the prevalence of pre-surgical NES range from 1.9% to 17.7% [16]. Patients with AN are by definition not obese and do not require bariatric procedures, however, AN-like presentations such as atypical anorexia nervosa (AAN) are becoming increasingly common in the general population and can present after bariatric surgery [8,16].

Studies suggest as well that people with EDs may be more likely to undergo weight-related cosmetic procedures such as liposuction compared to people without EDs. A study of 129 female ED inpatients found the rate of at least one lifetime cosmetic procedure to be twice that of women in the general population [12]. A population-based study also found 50% of young women with ED symptoms had an interest in liposuction and this was higher than in those without ED symptoms [13].

The wide range of prevalence of EDs in people undergoing surgical weight loss procedures is likely due to variable patient characteristics and ED assessment tools [17,19]. Furthermore, the existing literature has mainly studied the prevalence of BED in young females and there is a paucity of evidence regarding other populations including men and older adults and other ED features beyond binge eating [14,15,16,17,18,19,20,21,22,23,24,25]. To our knowledge, no studies have been performed on the prevalence of EDs and actual or intended bariatric surgery and cosmetic surgery in representative general population samples.

The primary aim of this study thus was to determine, in people with a current ED, prevalence estimates of (1) actual or intended bariatric surgery and (2) actual or intended cosmetic surgery including weight-related procedures. Secondary aims were to determine prevalence estimates of actual or intended (1) bariatric and (2) cosmetic surgery in people with a lifetime history of BN or BED, and also to investigate the associations between demographic features including age, sex, household income, body mass index (kg/m^2^; BMI), ED symptoms and the mental health-related quality of life (MHRQoL). We hypothesized that rates of actual or intended bariatric surgery would be higher in people with EDs than in people without an ED in the general population. No hypotheses were developed with regards to associations with demographic features, specific eating disorder symptoms or MHRQoL, as this was exploratory.

## 2. Materials and Methods

### 2.1. Study Design, Sampling and Weighting

Data for this study were obtained from the 2017 Health Omnibus Survey (HOS) conducted by Harrison Research, a 25-page interview with questions related to demographics and health. Participants from South Australian households took the HOS face-to-face between September and December 2017. Cities and towns were first stratified into metropolitan and country statistical areas (SAs) based on Australian Bureau of Statistics (ABS) Statistical Areas. A probability proportional to size procedure was used to select 398 metropolitan and 132 regional level 1 SAs (the smallest SAs by land area). Up to six attempts were made to interview eligible participants. A skip pattern of every fourth household was then used to select ten households within each level 1 SA and one interview with a person aged fifteen and over was conducted from each selected household. In instances where there was more than one person aged fifteen and over, the survey respondent was the person who had most recently had their birthday. Before commencing the interview, verbal consent was obtained. The interview was conducted face-to-face with interviewers reading questions aloud and using prompt cards where appropriate. Missing data were followed up by telephone and data were weighted by ABS 2016 Census data on age, sex, marital status, educational attainment, country of birth and household income.

### 2.2. Participants

**Figure 1:**
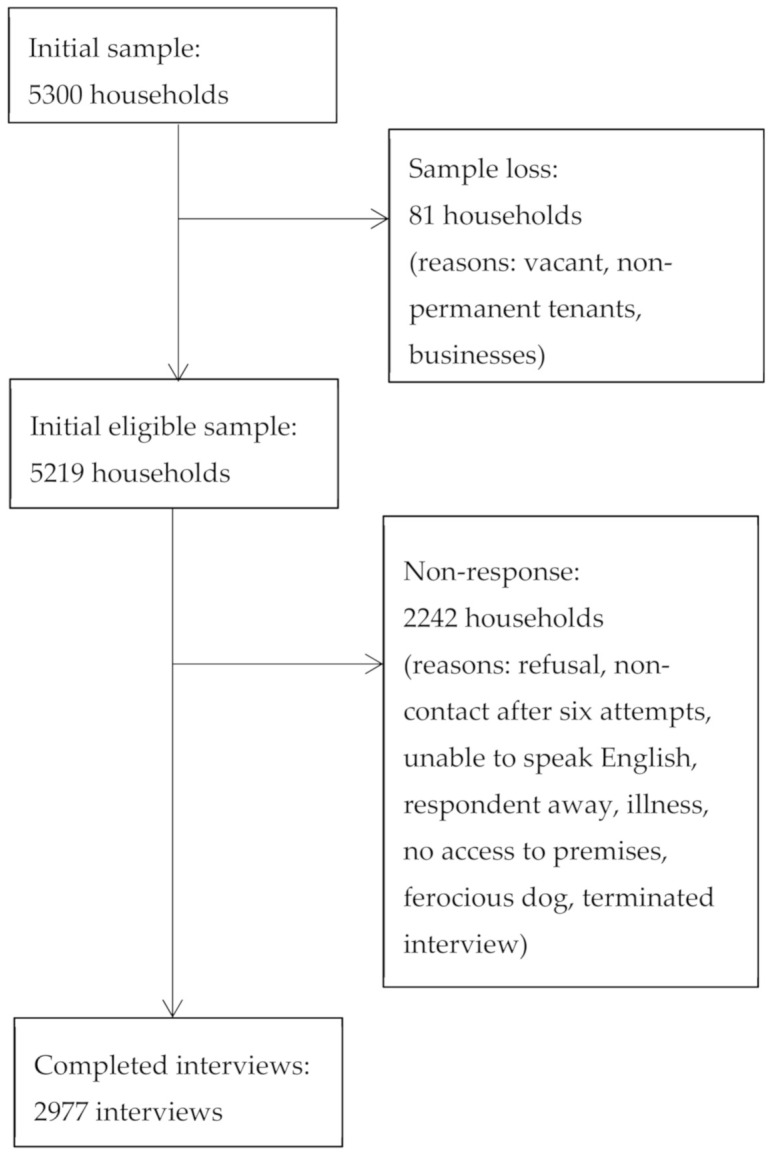
Participant selection.

From 5300 selected households, 2977 interviews were conducted. The participation rate, i.e., the number of completed interviews out of the initial eligible sample, was 65.3%. The overall response rate, i.e., the number of completed interviews out of the initial eligible sample minus households that were non-contactable after six attempts, was 57.0%. Refusal (25.3%) was the most common reason for non-response (see Figure 1. Participant Selection).

### 2.3. Interview Questions

#### 2.3.1. Demographic Questions

Demographic questions in this study assessed gender (male/female), age and household income.

#### 2.3.2. Eating Disorder (ED) Symptom Questions

Questions on ED symptoms were derived from the Eating Disorder Examination questionnaire so that diagnoses of BN, BED and other EDs could be assigned correctly [26]. They were asked about features as defined in the DSM-5 and included questions of clarification [8] (see Appendix A Box A1. The following features were assessed regarding the three months preceding the interview to diagnose current BN/BED and for any three-month period in the past to diagnose lifetime BN/BED: episodes of binge eating; distress associated with episodes of binge eating; dietary restriction and purging; and overvaluation of weight/body shape; along with BMI, electronically calculated from height and weight (self-reported).

Participants answered questions regarding overeating and loss of control to assess objective episodes of binge eating. Possible responses concerning distress associated with binge eating were: “not at all”, “yes—a little”, or “yes—a lot”, with “a lot” considered a response. Participants were included in the diagnostic category of BN based on DSM-5 criteria if they reported (1) BMI ≥ 18.5, (2) episodes of binge eating at least weekly, (3) weekly compensation by dietary restriction and/or purging and (4) extreme shape/weight overvaluation [8]. BED was defined more broadly than in the DSM-5 with reference to the 11th revision of the International Classification of Diseases (ICD-11) [27]. The ICD-11 criteria are more inclusive and align with the categories A, C, D and E for BED in the DSM-5 with the criteria for BN in the DSM-5. Participants were included in the diagnostic category of BED if they met the following criteria for at least three months: (1) BMI ≥18.5; (2) reporting recurrent binge eating episodes occurring at least weekly; (3) the episodes were associated with eating markedly more than usual or differently than usual; (4) episodes were associated with a subjective loss of control; and (5) marked distress with binge eating episodes. Further, in this study, the DSM-5 criteria for eating an objectively large amount of food were applied to the diagnosis of BED. Finally, participants who had regular binge eating or other ED symptoms (strict dieting or fasting in order to control weight/shape, and/or purging) and overvaluation, such as other specified feeding or eating disorder (OSFED) and unspecified feeding or eating disorder (UFED), but who did not meet the criteria for BN or BED, were categorized as “other ED”. Due to low base numbers, for the purpose of statistical analyses in this study, a variable ED was created of all AN, BN, BED, OSFED and UFED types. Lifetime diagnoses other than BN and BED were not derived.

#### 2.3.3. Bariatric and Cosmetic Surgery Questions

The following questions were asked of all participants: “Have you ever (in your lifetime) had or are you planning to have weight loss surgery? (e.g., stomach stapling, bariatric surgery, stomach or gastric sleeve or banding, gastric bypass)” and “Have you ever (in your lifetime) had or are you planning to have ‘cosmetic’ surgery such as liposuction or a similar weight related procedure?”.

#### 2.3.4. Mental Health Related Quality of Life

General mental health status was measured using the validated Short Form 12 Health-Related Quality of Life (SF12-HRQoL) tool administered as an interview [28,29]. This is a 12-item questionnaire that measures the limitations on function of physical and mental ill-health. It produces two weighted scales, a Physical Component Summary Scale (PCS) and a Mental Component Summary Scale (MCS). Each scale has a mean score of 50 and standard deviation of 10. Higher scores correlate with better health status. In previous Australian studies, it has been found to have robust psychometrics [29]. Raw scores were not provided for each SF12-HRQoL item so psychometrics such as internal consistency were not performed. The internal consistency of the previous HOS has been high [30].

### 2.4. Ethics

The 2017 HOS was approved by the University of Adelaide Human Research Ethics Committee (HREC). Ethics Approval ID: H-097-2010.

### 2.5. Data Analysis

Data were inspected for normality and cleaned. For the purposes of this study, all current main EDs (AN, BN, BED, OSFED) were grouped as one variable and lifetime BN and BED were grouped as one variable. Descriptive data are expressed in proportions with percentages, standard deviations and interquartile ranges. Chi-squared test (with Fishers exact test for small cell group sizes), t-test or the Mann–Whitney U statistic, as appropriate, were used to test for differences between groups and when exploring associations with demographic features, specific eating disorder symptoms or general mental health status.

## 3. Results

### 3.1. Bariatric Surgery

Sixty (2.0%, 95% C.I. 1.57–2.59) participants were intending to have or had received bariatric surgery. The proportion of women in this group was significantly higher than that of participants who had not planned or had a bariatric procedure (51.1%, *p* = 0.003) (Table 1). The mean age and median household income of participants did not significantly differ between the two groups. Those who reported actual/intended bariatric surgery had a significantly higher BMI (*p* < 0.001), higher weight/shape overvaluation (*p* < 0.001), more frequent binge eating (*p* < 0.001) and a lower MHRQoL (*p* = 0.002) when compared with those not reporting intended/actual bariatric surgery. Current EDs (*p* < 0.001), lifetime BN/BED (*p* < 0.001) and fasting/dieting (*p* < 0.001) were also significantly more prevalent in the bariatric surgery group.

### 3.2. Cosmetic Surgery

Thirty-two (1.1%, 95% C.I. 0.76–1.51) participants had planned or received cosmetic surgery (Table 2). There were significantly more females in this group than in the group who had not planned or had a cosmetic procedure (51.08%, *p* < 0.001). The mean age and median household income of participants did not significantly differ between the two groups. In the cosmetic surgery group, BMI was significantly higher (*p* < 0.001), MHRQoL was lower (*p* < 0.001), weight/shape overvaluation was higher (*p* = 0.002) and binge eating was more frequent (*p* = 0.035) when compared with participants without actual/intended cosmetic surgery. Current EDs (*p* = 0.002), lifetime BN/BED (*p* < 0.001) and fasting/dieting (*p* < 0.001) were also more prevalent in the cosmetic surgery group.

## 4. Discussion

To our knowledge, this is the first representative general population-based study investigating actual or intended bariatric or cosmetic surgery in people with current EDs. We found that participants who had or intended to have bariatric surgery were more likely to be female and have a higher BMI and poorer mental health status (as reflected in MHRQoL) compared to those who had not planned or received bariatric surgery. Those planning or having received bariatric surgery were also more likely to have a current ED, a lifetime history of BN or BED, practice dieting or fasting to control weight or shape, have high weight and/or body shape overvaluation and regular binge eating. The findings with regard to cosmetic surgery were similar. Participants planning or who had received cosmetic surgery were more likely to be female and have a higher BMI and poorer mental health status. Current EDs, lifetime history of BN or BED, dieting or fasting to control weight or shape, overvaluation of weight and/or body shape and binge eat regularly were more prevalent in the cosmetic surgery group.

The findings with regard to prevalence estimates of EDs and actual/intended bariatric surgery are in the upper-range of those reported in previous studies from clinical samples where very wide ranges are reported. The rate of current EDs in participants planning or receiving bariatric surgery was more than double that of those who had not planned or received the surgery (33.3% compared to 14.4%) and about double that of rates of EDs in the general Australian population (17.1%) [9]. The rate of lifetime BN/BED in the present study was 41.7% (over ten times the rate in the general Australian population) and this compares with clinical population samples of bariatric surgery candidates, which ranged from 6 to 64% [14,16,17,18,19]. Similarly, the rate of current EDs was 34.4% in those who were planned/actual recipients of cosmetic surgery compared to 14.6% in those not reporting this and 17.1% in the general Australian population. The rate of lifetime BN/BED was also 34.4% for planned/actual recipients of cosmetic surgery (up to ten times the rate in the general Australian population) compared to 6.1% in others. These figures are consistent with a previous study on ED inpatients, who were twice as likely to have had at least one previous cosmetic procedure than women in the general population [12]. They are also consistent with the population-based study that found that young women with ED symptoms were more likely to have an interest in cosmetic surgery than those without ED symptoms by as much as 50% [13].

This study found that the groups that had planned or received bariatric or cosmetic surgery had poorer MHRQoL than those who had not reported these procedures. This is consistent with most studies on bariatric surgery candidates with EDs. In one study, the rates of current and lifetime psychiatric disorders in surgical candidates were 55.5% and 72.6%, respectively [18]. A meta-analysis found that rates of mental health disorders were higher than the general population in pre-surgical groups and rates of depression consistently improved after surgery [21]. With regard to interest in cosmetic surgery in the general population, the findings have been more mixed. One study reported lower self-esteem and emotional lability to be associated with interest in liposuction [13]. However, another study of cosmetic surgery in ED inpatients found no significant difference in depressive symptomatology between groups [12].

The overrepresentation of women seeking both forms of surgery, as found in the present study, is well known [5,31,32]. It suggests that the reasons for seeking surgery may be less to do with health risks of high BMI and are more to do with societal regard for thinness accentuated for females [33]. In addition, women may be more likely to seek medical and surgical treatment for any reason and have been found to be more likely seek help for an ED [34,35].

### 4.1. Clinical Implications

The findings of this study suggest that health professionals working in bariatric and cosmetic surgery clinics should be aware of the overrepresentation of EDs and mental ill-health in their clientele. Screening for EDs and ED symptoms in candidates should be considered as identification and treatment of these mental health problems would improve the health of such attendees and may improve surgical outcomes and mental health status [36]. ED behaviors arise from the need to manage negative emotions and weight/shape concerns. These are heightened in the context of weight loss or cosmetic surgery where disordered behaviors such as irregular and binge eating if unchecked lead to adverse outcomes. Given the very high estimates found, such clinics may well benefit from staff with mental health expertise as people presenting for care may be reluctant to disclose ED symptoms on assessment questionnaires for fear they may not be offered the surgery [37,38]. However, the incorporation of a mental health assessment as a “routine” and the reassurance that mental health issues and EDs may be addressed prior to surgery can help to overcome this barrier to disclosure.

### 4.2. Strengths and Limitations

A notable strength of this study is the community-based sample, which reduces the bias seen in clinical populations. Furthermore, the sampling method and weighting of the data were used to ensure accurate representation of general population demographics. The interview-based survey increased confidence in respondents’ responses and allowed them to elaborate on their answers. The participation rate of the survey was also adequate, and participants were not aware of the specific ED and other content of the health survey prior to the interview. The major limitation of this study is that there was no distinction in the questionnaire between actual and intended surgery, or time since surgery. Data were also unavailable for type of planned/received surgical procedure, comorbidities of participants or compulsive exercise. Thus, people with an ED characterized by compulsive exercise alone would have been missed and thus the prevalence may be an underestimate. While data were available for any current ED, they were available only for lifetime BN/BED. The study was also limited in that the definition of lifetime BED was more broadly based on the 11th revision of the International Statistical Classification of Diseases and Related Health Problems (ICD-11) rather than the DSM-5. This however enabled adequate numbers of BED for statistical analyses. Diagnoses of EDs were also based on survey responses rather than being made by a clinician. Self-reporting of height and weight from which BMI was calculated may have been subject to response bias. Finally, a larger sample size would have allowed investigation of specific DSM-5 ED diagnoses.

### 4.3. Future Research

Future studies with larger population-based samples would enable investigation of individual ED diagnosis such as BN and BED or OSFED and thus distinguish between different types of EDs and between lifetime and current histories of EDs as well as between actual and intended bariatric and cosmetic surgery. This is important as it may be that specific EDs are more common than others (e.g., BED vs. BN) in people seeking such surgery. The impact of EDs on outcomes and merits of screening and treating prior to surgery is also important.

## 5. Conclusions

The estimated prevalence of actual or intended bariatric and cosmetic surgery is higher in people with a current ED and in people with a lifetime history of BN or BED. People who have had or plan to undergo bariatric and cosmetic surgery are more likely to be female, have a higher BMI, poorer MHRQoL and a current or lifetime history of ED features. These findings have implications for surgical clinics. We support screening for and treating EDs in surgical candidates as this may help identify patients who need additional input before and after surgery, as well as improve their surgical outcomes, mental health and physical health. The present research would be informed by future studies that use larger population-based samples and distinguish between current and previous histories of EDs and different types of EDs.

## Figures and Tables

**Table 1:** Demographic, mental health and eating disorder (ED) features of people who had planned or received bariatric surgery (*n* = 60) and not planning or having received bariatric surgery (*n* = 2917).

	Bariatric Surgery Status		Statistic	
	Planned/Received	Not Planned/Received		
	*n* (%)		χ^2^ (df = 1)	*p*
Female *	43 (70.49)	1473 (51.11)	8.984	0.003
Current ED	20 (33.33)	416 (14.44)	16.616	<0.001
Lifetime BN/BED	25 (41.67)	164 (5.69)	126.55	<0.001
Fasting/Dieting	10 (16.67)	136 (4.73)	17.74	<0.001
	Mean (SD) *n*		t (df)	*p*
Age (years)	48.42 (14.29) 60	47.54 (19.14) 2882	0.356 (2940)	0.722
BMI (kg/m^2^)	35.92 (8.06) 55	26.99 (5.70) 2640	11.36 (2693)	<0.001
MHRQoL (SF-12 MCS)	48.28 (12.48) 60	52.02 (9.19) 2869	−3.10 (2927)	0.002
	Median (IQR) *n*		Mann–Whitney U-Z	*p*
Annual household income band ($Aus)	4 (2–5) 50	4 (2–5) 2123	−0.576	0.564
Overvaluation of weight/shape	4 (3.7–6) 60	3 (1–5) 2882	−5.463	<0.001
Binge eating	1 (1–2.52) 60	1 (1–1) 2882	−4.286	<0.001

χ^2^ = chi-square, * = Fisher exact test where applicable, ED = eating disorder, BN = bulimia nervosa, BED = binge eating disorder. MHRQoL = mental health-related quality of life. SF12 MCS = Short Form 12 Mental Component Summary score. $Aus = Australian dollar income bands: 1. <AUD 20,000, 2. AUD 20,001–AUD 40,000, 3. AUD 40,001–AUD 60,000, 4. AUD 60,001–AUD 100,000, 5. AUD 100,001–AUD 140,000, 6. >AUD 140,001.

**Table 2:** Demographic, mental health and ED features of people who had planned or received cosmetic surgery (*n* = 32) and not planning or having received cosmetic surgery (*n* = 2945).

	Cosmetic Surgery Status		Statistic	
	Planned/Received	Not Planned/Received		
	*n* (%)		χ^2^ (df = 1)	*p*
Female	29 (90.63)	1486 (51.08)	19.81	<0.001 *
Current ED	11 (34.38)	426 (14.64)	9.74	0.002
Lifetime BN/BED	11 (34.38)	177 (6.08)	42.34	<0.001
Fasting/Dieting	9 (28.13)	137 (4.72)	36.70	<0.001
	Mean (SD) *n*		t (df)	*p*
Age (years)	45.69 (13.28) 32	47.57 (19.10) 2909	−0.557 (2939)	0.577
BMI (kg/m^2^)	31.27 (7.22) 30	27.13 (5.86) 2664	3.857 (2693)	<0.001
MHRQoL (SF-12 MCS)	46.22 (11.46) 32	52.01 (9.24) 2896	−3.523 (2926)	<0.001
	Median (IQR) *n*		Mann–Whitney U-Z	*p*
Annual household income band ($Aus)	3 (2–4) 31	4 (2–5) 2142	−1.49	0.137
Overvaluation of weight/shape	5 (3–6) 32	3 (3–5) 2825	−3.14	0.002
Binge eating	1 (1–2) 32	1 (1–1) 2829	−2.11	0.035

χ^2^ = chi-square, * Fisher exact test where applicable, ED = eating disorder, BN = bulimia nervosa, BED = binge eating disorder. MHRQoL = mental health-related quality of life. SF12 MCS = Short Form 12 Mental Component Summary Score. $Aus = Australian dollar income bands: 1. <AUD 20,000, 2. AUD 20,001–AUD 40,000, 3. AUD 40,001–AUD 60,000, 4. AUD 60,001–AUD 100,000, 5. AUD 100,001–AUD 140,000, 6. >AUD 140,001.

## Appendix A

Box A1 Weight control questions. I would now like to ask you about episodes of overeating. By overeating or binge eating, I mean eating an unusually large amount of food in one go and at the time feeling that your eating was out of control. *Respondent could not prevent themselves from overeating, or could not stop eating once they had started.* Over the past 3 months, how often have you overeaten? (Responses were on a set list of ‘not at all’, ‘less than weekly’, ‘once a week’, or ‘two or more times a week’). Is the binge or overeating you experience usually associated with distress? (Responses were on a set list of ‘Not at all’, ‘Yes—little’, or ‘Yes—a lot’). Is the binge or overeating you experience usually associated with? (Responses were on a set list of ‘Eating much more rapidly than normal’, ‘Eating until feeling uncomfortably full’, ‘Eating large amounts of food when not feeling physically hungry’, ‘Eating alone because you are embarrassed about how much you are eating’, ‘Feeling disgusted, guilty or very depressed after eating’, ‘None of these’). Over the past three months typically (on average) how many times a week have you overeaten? Over the past 3 months have you felt your eating was out of control when others might not agree the amount of food was unusually large (e.g., 2–3 pieces of bread)? Would you say… *Note ‘Out of control’ refers to respondent could not prevent themselves from overeating, or they could not stop eating once they had started on smaller or more usual amounts of food.* (Responses were on a set list of ‘not at all’, ‘less than weekly’, ‘once a week’, or ‘two or more times a week’). Is this smaller overeating you experience usually associated with distress? The next three questions are about various weight-control methods some people use. Over the past three months have you regularly used, that is at least once a week, any of the following: laxatives, diuretics (water tabelts), made yourself sick, in order to control your shape or weight? (Responses were on a set list of ‘Yes’ or ‘No’). Over the past 3 months, have you regularly done any of the following: gone on a very strict diet, or eaten hardly anything at all for a time, in order to control your shape or weight? At least once weekly, or recurrently during the three months. (Responses were on a set list of ‘Yes’ or ‘No’). On a scale of 0–6, where 0 is Not at all important and 6 is Extremely or the most important issue. How important an issue has your weight and/or your shape been to how you think about (judge or view) yourself as a person in the past three months? *It has been a really important issue to them, their self-esteem or their self-confidence*. In the past 3 months have you had any episodes of night eating? By night eating I mean waking from sleep and eating (i.e., you were not sleep walking and eating, you were awake), OR episodes of eating a very large amount after your evening meal. (*This does not include eating at night because of social or other circumstances* e.g., *you are travelling overseas on a night flight or because of work shifts*) Would you say you are night eating… (Responses were on a set list of ‘not at all’, ‘less than weekly’, ‘once a week’, or ‘two or more times a week’). Is the night eating you experience usually associated with distress? (Responses were on a set list of ‘Not at all’, Yes—little’, or ‘Yes—a lot’). Have you ever (in your lifetime) regularly used or been prescribed drugs to reduce your appetite and/or reduce overeating or binge eating? (e.g., Duromine, an amphetamine, topiramate, Topamax, orlistat, Xenical) (Responses were on a set list of ‘Yes’ or ‘No’). Have you ever (in your lifetime) had or are you planning to have weight loss surgery? (e.g., stomach stapling, bariatric surgery, stomach or gastric sleeve or banding, gastric bypass) (Responses were on a set list of ‘Not at all’, ‘Yes—little’, or ‘Yes—a lot’). Have you ever (in your lifetime) had or are you planning to have ‘cosmetic’ surgery such as liposuction or a similar weight related procedure? (Responses were on a set list of ‘Not at all’, ‘Yes—little’, or ‘Yes—a lot’). These questions are similar to questions you have already answered about your eating and weight but these are in reference to your past. In the past, have you ever had a period of time when episodes of overeating were occurring, on average, at least once a week for 3 months? By overeating, or binge eating, I mean eating an unusually large amount of food in one go and at the time feeling that your eating was out of control. *Respondent could not prevent themselves from overeating, or could not stop eating once they had started.* (Responses were on a set list of ‘Yes’ or ‘No’). Were the binge or overeating episodes you experienced usually associated with distress? (Responses were on a set list of ‘Not at all’, ‘Yes—little’, or ‘Yes—a lot’). On a scale of 0–6, where 0 is No importance and 6 is Extremely important or the most important issue. How important an issue was your weight and/or your shape to how you thought about (judged or viewed) yourself as a person during the period/time these episodes of overeating were occurring? Was it a really important issue to them, their self-esteem or self-confidence? During these episodes of overeating/binge eating, did you regularly, that is at least once a week for at least 3 months, use a very strict diet, or eat hardly anything at all for a time, and/or exercise vigorously, in order to control your shape or weight? (Responses were on a set list of ‘Yes’ or ‘No’). During these episodes of overeating/binge eating, did you regularly, that is at least once a week for at least three months, use any of the following: laxatives, diuretics (water tablets), made yourself sick, in order to control your shape or weight? (Responses were on a set list of ‘Yes’ or ‘No’). In the past, during these episodes, what was your estimated weight, height and age? If more than one episode, choose lowest weight if weight varied, or (if not possible to estimate lowest weight period) choose the most typical/average weight during any single episode. (Age and height is from the same period the respondent selected for weight).
